# The role of neck adipose tissue in lymph node metastasis of head and neck cancer

**DOI:** 10.3389/fonc.2024.1390824

**Published:** 2024-05-10

**Authors:** Yiqi Pan, Ying Xu, Cui Fan, Xiangwan Miao, Yilin Shen, Quan Wang, Jichang Wu, Haixia Hu, Hao Wang, Mingliang Xiang, Bin Ye

**Affiliations:** Department of Otolaryngology & Head and Neck Surgery, Ruijin Hospital Affiliated to Shanghai Jiaotong University School of Medicine, Shanghai, China

**Keywords:** adipose tissue, head and neck cancer, lymph node metastasis, tumor microenvironment, lymphangiogenesis

## Abstract

Previous studies indicated that adipose tissue significantly influences cancer invasion and lymphatic metastasis. However, the impact of neck adipose tissue (NAT) on lymph node metastasis associated with head and neck cancer remains ambiguous. Here, we systematically assess the classification and measurement criteria of NAT and evaluate the association of adipose tissue and cancer-associated adipocytes with head and neck cancer. We delve into the potential mechanisms by which NAT facilitate cervical lymph node metastasis in head and neck cancer, particularly through the secretion of adipokines such as leptin, adiponectin, and Interleukin-6. Our aim is to elucidate the role of NAT in the progression and metastasis of head and neck cancer, offering new insights into prevention and treatment.

## 1 Introduction

Cervical lymph nodes metastasis (LNM) is crucial in the clinical staging of head and neck cancer (HNC), and it serves as a vital indicator for assessing the progression and prognosis (1). Although adipose tissue (AT) is the predominant tissue surrounding cervical LNM, its relationship with LNM in HNC remains elusive. Previous studies have shown that breast cancer and prostate cancers are surrounded by abundant AT, forming a unique microenvironment between AT and cancer cells (2, 3). There exists a same crosstalk between cancer cells and adipocytes in HNC. This interplay continuously alters the tumor microenvironment, thus leading to the formation of specialized AT. AT can induce metabolic reprogramming in cancer, facilitating the uptake of free fatty acids and glycerol from adipocytes. This uptake serves as an energy source for oxidative phosphorylation in mitochondria, resulting in an “anti-Warburg effect” that enhances the invasion and metastasis of cancers such as breast and prostate cancers (2–4). AT releases higher levels of adipose-derived cytokines, such as leptin, Interleukin-6 (IL-6), and CC-chemokine ligand 5 (CCL5), promoting cancer proliferation and invasion (2). Esposito et al. identified a notable association between positive CCL5 staining in peritumoral adipocytes and LNM in breast cancer (5). Further, adipocytes in breast cancer rely on the fatty acid synthase ACSL3(acyl-CoA synthetase long-chain family member 3) to release MUFA (oleic acid), enabling cancer to resist ferroptosis (6). In prostate cancer, AT plays a role in recruiting immunosuppressive cells, modifying the extracellular matrix, supporting neovascularization, and inducing malignant tumor invasion (7). Yousuke Shimizu et al. discovered the existence of 2% LNM in the prostatic anterior fat pad of prostate cancer patients. Consequently, Urology guidelines recommend the routine removal of prostatic anterior fat pad during radical prostatectomy to minimize the risk of residual tumor tissue (8). It is evident that AT significantly influences LNM in some malignant tumors such as breast and prostate cancers.

Recent studies have identified an indirect link between AT and the invasion and metastasis of HNC (9, 10). Studies focusing on thyroid cancer (11), nasopharyngeal carcinoma (12), and oral squamous cell carcinoma (13) have demonstrated associations between body mass index (BMI) and incidence rate, aggressive pathological features, and unfavorable clinical outcomes. Lymphatic metastasis serves as a primary route for the local metastasis of HNC. AT, the principal energy source in the tumor microenvironment, facilitates lymphangiogenesis (14). Additionally, overwhelming evidence supports the notion that AT is an endocrine tissue that can secrete a variety of adipokines, such as leptin (15), adiponectin (16), and IL-6 (17), which also contribute to cancer invasion (15). Therefore, we analyzed and summarized the correlation between NAT and lymph node metastasis in HNC. We started by summarizing the current methodologies for quantifying NAT. Then, we tried to research the associations between NAT and LNM in HNC. Finally, we analyzed the mechanisms by which NAT might promote LNM. Our aim is to identify potential NAT risk factors for LNM in HNC, ultimately improving the prognosis of patients with HNC.

### 1.1 Definition, classification, and function of adipose tissue

AT, a specialized connective tissue, predominantly consists of adipocytes (18). Beyond adipocytes, it also comprises adipose-derived stem cells, preadipocytes, fibroblasts, lymphocytes, macrophages, and vascular endothelial cells (19). It is essential for mechanical support, thermoregulation, energy storage and release, appetite, and immune regulation (18). BMI is calculated by the formula: weight divided by height squared (kg/m²), which serves as an indirect indicator of overall adiposity (20).

There are three principal types of AT, namely, white adipose tissue (WAT), brown adipose tissue (BAT), and beige adipose tissue. WAT is characterized by large unilocular lipid droplets and a limited number of mitochondria (21). WAT is the most abundant type in the adult neck (22). WAT primarily regulates the storage and release of energy to cater to the needs of various tissues (23). White adipocytes can be converted to thermogenic beige adipocytes following stimuli such as exercise or cold exposure, a process termed “browning of WAT” (24). Beige adipocytes are thermogenically active, bear a morphological and biochemical resemblance to BAT, and include multilocular lipid droplets and abundant mitochondria (25).

BAT is distinguished by small multilocular lipid droplets and a profusion of mitochondria rich in cytochrome content (21). A series of studies using 18F-FDG-PET/CT detection found that the most common location of BAT in the adult neck is in the frontal aspect, superficial and lateral to the sternocleidomastoid muscle, and supraclavicular regions (26, 27). Although BAT constitutes a minor portion of body mass, it is pivotal for non-shivering heat production during cold exposure (18). Recent studies have revealed intriguing links between BAT and cancer proliferation. Takahiro Seki et al. observed that cold environment was found to activated substantial BAT in the mice, leading to inhibited energy uptake and consequent tumor cell apoptosis (28).

## 2 Relationship between BMI, NAT and lymph node metastasis in HNC

### 2.1 Relationship between BMI, NAT and lymph node metastasis in thyroid cancer

NC can serve as a direct indicator of NAT accumulation around the respiratory tract or within the cervical subcutaneous AT layer, while BMI provides an indirect assessment of NAT. Many studies have delved into the prognostic value of NC and BMI in thyroid cancer. Excluding patients with a tumor size greater than 2 cm, Kim et al. found that male patients with lateral LNM had a notably larger NC compared with those without metastasis. Thus, NC emerged as a predictor of cervical LNM in male patients with thyroid cancer (29). A retrospective cohort study involving 796 patients diagnosed with early-stage papillary thyroid cancer found that when BMI was ≥18.5 kg/m², the average number of LNM in the central and lateral cervical regions increased proportionately with BMI. For overweight patients, the incidence of central and lateral cervical LNM was 55.3% and 37.9%, respectively. By contrast, these figures for the obese group were 55.9% and 45.3%, respectively (30). Similarly, Li et al. demonstrated that obese patients with papillary thyroid cancer had a significantly higher incidence of metastasis to the central and lateral lymph nodes compared with their normal-weight counterparts (31). By contrast, Zhao et al. found that their findings did not reveal any significant differences in cervical LNM across different BMI categories (32). Numerous studies have demonstrated that overall AT and NAT facilitate the process of LNM in thyroid cancer. However, disparities between individual studies arise from variations in sample size, ethnic background, and the inherent limitation of BMI (its inability to distinguish between muscle and AT or to quantify specific AT compartments). Although there is a strong correlation between BMI and NAT, there is insufficient evidence to directly replace association between BMI and HNC with that between NAT and HNC.

### 2.2 Relationship between BMI, NAT and other HNC

BMI serves as a significant indicator of NAT. In the context of oral cancer, Bao et al. analyzed BMI data from 1,395 oral cancer patients between 2007 and 2018. They observed that underweight patients exhibited inferior survival outcomes (HR = 1.585; 95% CI: 1.207–2.082) (13). Choi et al. employed contrast-enhanced CT scans of the neck (from the anterior superior of the hyoid to the third cervical spine and inferiorly to the first rib) in 79 patients with various HNC. Using the 3D slicer tool, they measured NAT volume changes both pre- and post-radiotherapy over one year. Their findings indicated that patients with low NAT volume before and after treatment had poorer overall survival rates. Further, significant weight loss during treatment was also linked to diminished overall and recurrence-free survival rates (33). An increased NAT volume appears to improve the prognosis of patients with HNC. This improvement might be attributed to the protective role of AT in helping patients withstand the side effects of radiotherapy and the nutritional challenges associated with cancer. Expanding on this, Huang et al. studied 400 stage III or IVa nasopharyngeal carcinoma patients. Their research probed the correlation between pretreatment BMI and clinical outcomes in patients undergoing chemoradiotherapy. The results revealed 5-year failure-free survival rates of 44%, 61%, 68%, and 73%, and 5-year overall survival rates of 51%, 68%, 80%, and 72% for underweight, normal weight, overweight, and obese groups, respectively (34). They postulated that an adequate volume of AT could potentially ameliorate the adverse effects of chemoradiotherapy in advanced nasopharyngeal carcinoma cases and counteract cachexia in cancer patients. Similarly, our research group has previously demonstrated that adipose tissue and lipid metabolism related factors exhibited a regulatory influence on the process of LNM and prognosis in patients with HNC (35, 36). However, given the limited research on the interplay between NAT and HNC, further studies are essential to ascertain the exact impact of NAT on prognosis and the potential mechanisms by which AT might promote cervical LNM in HNC.

## 3 Mechanism of adipose tissue promoting cervical lymph node metastasis of HNC

### 3.1 Lymphangiogenesis enhanced by adipose tissue

Lymphatic vessels in the human body play a significant role in lipid transport and absorption. Peter et al. reported that the expression of the fatty acid β-oxidation (FAO) pathway was markedly elevated in lymphatic vessels compared with other vessel types. Further investigation showed that by utilizing fatty acids for β-oxidation, lymphatic endothelial cells enhanced the expression of the lymphangiogenic factor-prox1, thus facilitating the formation of new lymphatic channels (37). The vascular endothelial growth factor-C (VEGF-C) stands out as a potent lymphangiogenic factor. Adipose-derived stem cells (ADSCs) are known to secrete growth factors and exosomes, thereby modulating the tumor microenvironment. A study carried out in 2018 unveiled that following VEGF-C treatment, ADSCs-secreted miR-132 was transferred to lymphatic endothelial cells via exosomes. The uptake of miR-132 by these cells stimulated their proliferation, migration, and formation of lymphatic channels. This discovery underscores the regulatory role of ADSCs exosomes in VEGF-C-mediated lymphangiogenesis (38). Collectively, these insights highlight the importance of AT in lymphatic vessel development and functionality. When primary tumors are present in the head and neck regions, NAT might facilitate lymphatic metastasis of these tumors by regulating lymphatic vessel.

### 3.2 Cancer-associated adipocytes

Adipocytes that interact with cancer cells are termed “cancer-associated adipocytes” (4). The idea that adipocytes might influence tumor progression was initially suggested by Spector et al. In 2003, Puneeth et al. discovered that adipocytes surrounding breast cancer tissues promoted tumor progression. This promotion was achieved by the secretion of collagen VI, which induced an anti-apoptotic transcriptional program and stabilized proto-oncogenes in tumor cells (39). Subsequently, Dirat et al. revealed that breast cancer cells exhibited increased invasiveness when co-cultivated with mature adipocytes. Further, the number of lung metastases was enhanced in mice injected with adipocytes co-cultivated with 4T1 tumor cells compared with mice injected with 4T1 cells alone. Intriguingly, when co-cultured with breast tumor cells, mature adipocytes showed a marked reduction in the number and size of lipid droplets and a decreased expression of adipocyte differentiation markers such as hormone-sensitive triglyceride lipase (HSL), resistin, and adiponectin. By contrast, upregulation of the expression of proinflammatory cytokines (e.g., IL6, IL1β, TNFα) and matrix remodeling proteins (e.g., MMP-11, PAI-1) was observed. In various solid tumors such as breast cancer (4), prostate cancer (7), melanoma (40), and colorectal cancer (41), the invasion of tumor cells into the surrounding AT is linked to a profound reduction of lipid in adipocytes. Nieman et al. noted that ovarian cancer preferentially metastasizes to the omentum, which is rich in adipocytes. They further found that co-culturing adipocytes with ovarian cancer cells led to a direct lipid transfer from adipocytes to the cancer cells. This process allowed cancer cells to utilize fatty acids by β-oxidation (42). In addition, cancer-associated adipocytes have been found to release high levels of cytokines and growth factors such as IL-6, CCL2, CCL5, IL1β, TNFα, and VEGF, which collectively contribute to enhanced tumor cell proliferation, invasion, and angiogenesis (43). Consequently, the interaction between cancer-associated adipocytes and cancer cells in the tumor microenvironment serves to bolster the survival, proliferation, and metastatic potential of the cancer through direct lipid exchange or adipokine secretion (44).

### 3.3 Adipokine in the tumor microenvironment promotes lymph node metastasis in HNC

#### 3.3.1 Leptin

Leptin is a product encoded by the LEP gene on human chromosome 7. It is a 16 kDa adipokine synthesized and secreted by adipocytes, primarily playing a crucial role in regulating energy metabolism and promoting cell proliferation. Both leptin and its receptor are highly expressed in thyroid cancer, salivary gland carcinoma, oral squamous cell carcinoma, and laryngeal cancer. Further, the expression of leptin and its receptor is positively correlated with cancer invasiveness indicators including tumor size and LNM (45–49). Cheng et al. assessed the levels of leptin and its receptor in 49 primary tumors and 15 LNM using immunohistochemistry. They discovered that leptin and its receptor were expressed in 37% and 51% of papillary thyroid carcinomas, respectively. The co-expression of leptin and its receptor in primary tumors was associated with a higher likelihood of LNM (50). Leptin can stimulate tumor cells invasion and inhibit tumor cells apoptosis. Eliane et al. found that in SCC-9 and SCC-4 oral squamous cell lines, leptin promoted the expression of genes related to angiogenesis and invasiveness such as E-cadherin, Col1A1, MMP2, and MMP9, thereby enhancing cell proliferation and invasiveness (49). Further, leptin can enhance the migration of thyroid cancer cells through the PI3K/AKT and MEK/ERK signaling pathways (51). Through an *in vitro* study, Shahab et al. determined that overexpression of the leptin receptor can inhibit apoptosis by upregulating BCl-XL and XIAP (anti-apoptotic genes) (47).

#### 3.3.2 Adiponectin

Adiponectin is a primary adipokine secreted by AT that can also be produced by cardiomyocytes, skeletal muscle cells, and lymphocytes (52). Adiponectin belongs to the complement factor C1q-like protein superfamily. Adiponectin primarily functions in regulating glucose metabolism and stimulating FAO (53). Recently, a strong inverse correlation was shown between adiponectin levels and the incidence of various malignant tumors, such as colorectal cancer, breast cancer, prostate cancer, leukemia, and endometrial cancer. Adiponectin is also considered a potent anticancer factor that inhibits cancer growth. In endometrial cancer, adiponectin activates AMPK and downregulates Bcl-2 and MMP-9 expression, consequently inhibiting the invasion of tumor cells and promoting tumor cell apoptosis (54). However, research on the association between adiponectin and HNC is limited. Nicholas et al. found a significant independent negative correlation between circulating adiponectin levels and the risk of thyroid cancer (55). Cheng et al. determined that AdipoR1 was expressed in 27% of primary malignant tumors, while AdipoR2 was found in 47% of primary malignant tumors via immunohistochemical staining of 49 thyroid tumor samples and metastatic lymph nodes. In addition, negative expression of both adiponectin receptors was significantly correlated with extrathyroidal invasion, multicentricity, and higher TNM staging (56). Ersilia et al. discovered that adiponectin inhibited the proliferation of papillary thyroid cancer cell lines (BCPAP and K1) and anaplastic thyroid cancer cell lines (CAL62). Current research suggests that adiponectin exerts its effect by binding to its receptors and regulating AKT/mTOR/PI3K and MAPK signaling pathways, which are associated with cell proliferation and energy modulation, thereby inhibiting the activity and growth of thyroid cancer (16). Evidently, adiponectin serves as a protective adipokine against HNC. Therefore, reduced levels of adiponectin in obese individuals can potentially promote the onset of HNC.

#### 3.3.3 Interleukin-6

IL-6 is a multifunctional cytokine that plays a crucial role in the broad biological activity of cancer cells. IL-6 is involved in immune modulation and tumorigenesis. In pathological conditions of obesity and cancer, IL-6 levels secreted by adipocytes are significantly increased (17). Nandita et al. found that serum IL-6 levels positively correlated with tumor size, extrathyroidal invasion, and distant metastasis in papillary thyroid carcinoma patients (17). In prostate cancer (57), breast cancer (58), ovarian cancer (59), non-small cell lung cancer (60), and endometrial cancer (61), IL-6 also had a correlation with clinical progression of the cancer. Numerous studies suggest that IL-6 can promote tumor invasion, inhibit tumor cell death, and facilitate tumor cell immune evasion through various mechanisms. IL-6 binds to a specific binding receptor on the cell membrane (IL-6R), leading to activation of the JAK/STAT3 pathway and promoting thyroid tumor cell invasion (62). Similarly, Mingyu et al. collected specimens from normal tissues, vocal cord leukoplakia, and HNC. they found that levels of IL-6 were higher than in normal epithelium. It was discovered that IL-6 transcriptionally activates xCT, a key amino acid antiporter, via the JAK2/STAT3 signaling pathway. The upregulation of xCT induces ferroptosis resistance and tumor progression, suggesting IL-6 as a novel oncogenic ferroptosis inhibitor (63).

#### 3.3.4 Other substances

Extracellular vesicles serve as a critical conduit for communication between adipocytes and tumor cells. These vesicles transport proteins and fatty acids related to lipid metabolism. Once internalized by tumor cells, they enhance FAO (14). Adipocytes cultured in high-fat conditions exhibit an increased secretion of extracellular vesicles. The fatty acids from these vesicles accumulate in the lipid droplets of cancer cells and are subsequently released during fat autophagy, further driving FAO (14). Further, cancer cells can release extracellular vesicles that stimulate lipolysis in adipocytes (44). For example, extracellular vesicles from lung cancer cells were found to be enriched with IL-6, which triggers lipolysis in adipocytes by activating the STAT3 pathway (64). Beyond this, adipose tissue releases other adipokines including interleukin-8 (65), IGFBP-2 (66), β-hydroxybutyrate (67), CD36 (35), FASN (35) and IGF-1 (68), which can influence tumor invasion.

## 4 Conclusion

Given the association of breast cancer, prostate cancer, and other malignant tumors with surrounding AT, as well as studies correlating NC and BMI with LNM in HNC, it is evident that NAT foster cervical LNM in HNC. On the one hand, NAT can directly fuel tumors and nascent lymphatic vessels through FAO. In addition, it might secrete adipokines or extracellular vesicles that modulate tumor growth or alter the microenvironment around the malignant tumor, thereby influencing malignant tumor invasion and migration. On the other hand, HNC can induce the transformation of typical adipocytes in the neck to cancer-associated adipocytes. This can shift the metabolic expression patterns of HNC, creating a feedback loop that fosters growth, migration, and LNM.

Currently, the bulk of research centers on the association between BMI and HNC, with limited epidemiological and foundational studies directly linking NAT to HNC. There is no universally accepted methodology for quantifying NAT. Future research should prioritize investigating the connection between NAT and various HNC, delineate the alterations in submental NAT instigated by HNC and discern the role of NAT in the progression of HNC. Delving into the mechanisms by which NAT drives HNC and LNM could provide novel insights and strategies for the diagnosis and management of HNC.

## Author contributions

YP: Writing – original draft, Writing – review & editing. YX: Writing – original draft. CF: Writing – review & editing. XM: Writing – review & editing. YS: Writing – review & editing. QW: Writing – review & editing. JW: Writing – review & editing. HH: Writing – review & editing. HW: Writing – review & editing. MX: Writing – review & editing. BY: Writing – original draft, Writing – review & editing.
